# Could Methotrexate Be an Option for Managing Ectopic Pregnancies in Women With an Initial High β-Human Chorionic Gonadotropin Level?

**DOI:** 10.7759/cureus.96079

**Published:** 2025-11-04

**Authors:** Sherin Hassaballa, Ahmed Abdelmohsen, Paul Armstrong, Nayantara Bijral

**Affiliations:** 1 Obstetrics and Gynaecology, William Harvey Hospital, East Kent Hospitals University NHS Foundation Trust, Ashford, GBR

**Keywords:** ectopic pregnancy, failure rate and expected resolution time, high initial human chorionic gonadotrophin level, methotrexate medical management, persistent ectopic mass

## Abstract

Ectopic pregnancy is a common clinical problem with a formal evidence-based practice guideline. In this case, the patient declined the recommended standard management following counselling. In this report, we present the case management, time to resolution, and patient perspective for refusal of standard treatment.

We reviewed the recommendations of the National Institute for Health and Care Excellence (NICE) and American College of Obstetricians and Gynecologists (ACOG) guidelines with the supporting evidence, along with other published studies evaluating the effectiveness of methotrexate with varying initial levels of β-human chorionic gonadotropin (β-hCG). Informed patient choice, as a part of modern medical practice, is addressed with reference to novel practices in obstetrics. We questioned whether methotrexate could be an option for these women following thorough counselling.

The significance of a persistent ectopic mass after clinical resolution is highlighted as an area that requires further research to evaluate the benefits and risks of this treatment option.

## Introduction

In the UK, the rate of ectopic pregnancy is 11 per 1,000 pregnancies, with around 11,000 ectopic pregnancies diagnosed each year [1]. There were 12 maternal deaths from ectopic pregnancy in 2021-2022, and assessors felt that improvements to care would have made a difference to 75% of these women [2].

In the US, ectopic pregnancy occurs in approximately 2% of all pregnancies, with ruptured ectopic pregnancies accounting for 2.7% of all pregnancy-related deaths [3].

Options for treatment include medical, surgical, or expectant management. The choice of management of ectopic pregnancy depends upon multiple factors, including clinical presentation, haemodynamic stability, ultrasound scan features, and serum β-human chorionic gonadotropin (β-hCG) level [1]. Surgical management was historically offered as the treatment of choice, which still applies in the situation of haemodynamic instability and with evidence of rupture. Medical management has received increased attention after it was first introduced in 1982 [4]. Methotrexate is now the most commonly used agent for the medical treatment of women who are in a clinically stable condition and meet the strict follow-up requirements [5]. Methotrexate is a folic acid antagonist that inhibits DNA synthesis, cell repair, and replication, thus affecting the rapidly proliferating tissue such as trophoblastic tissue [3].

As the most common life-threatening condition in gynaecology, determining the safe thresholds when offering medical management has been the focus of many studies over the years. The correlation between failure rates of medical management and subsequently the risk of ectopic rupture, and factors like the size of the mass on ultrasound, the initial β-hCG level, and the presence of fetal heart activity, was the drive to set thresholds for offering medical management. Initial pre-treatment β-hCG level was found to be the most important predictor for failure by many studies [6,7]. However, the threshold value that best predicts success or failure, with the exact β-hCG level over which medical treatment is more likely to fail, is not known [8].

## Case presentation

**Figure 1:**
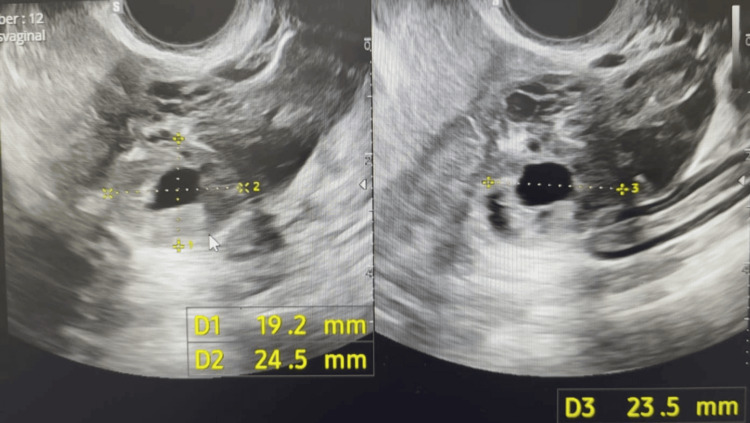
An echogenic avascular homogeneous mass with an anechoic centre was noted measuring 19 x 24 x 23 mm adjacent to the ovary.

A 40-year-old, six-week-pregnant female (G1P0) presented to the accident and emergency department (A&E) with increasing right-sided iliac fossa pain and light red vaginal bleeding for three days. On presentation, she was haemodynamically stable. Her serum β-hCG level was 12,867 IU/L. She was diagnosed with a right tubal ectopic pregnancy after a transvaginal scan. The scan showed an echogenic avascular homogeneous mass with anechoic centre measuring 19 x 24 x 23 mm adjacent to the ovary, and no free fluid was seen (Figure 1).

Her symptoms had started two weeks earlier with brownish vaginal discharge and mild pelvic pain, soon after having a positive home pregnancy test. She had been offered a β-hCG check and transvaginal scan when she sought medical advice. Her β-hCG was 399 IU/I, though she declined a scan at that time.

Our patient had a history of primary male-factor infertility. She had regular cycles lasting five days every 28 days, was a non-smoker, with no history of previous surgery or any medical history of concern apart from anxiety disorder.

Once the ectopic pregnancy was diagnosed with ultrasound and a β-hCG of 12,867 IU/L, the patient was admitted, and a discussion regarding the surgical management of the ectopic pregnancy was conducted. Increased failure rate of medical management with higher initial β-hCG, the risk of ruptured ectopic pregnancy as a serious outcome, and the need for emergency surgery were discussed. The patient declined surgery, as removing the fallopian tube and the chance of this affecting her fertility was not acceptable to her. She decided to have methotrexate as a treatment for her ectopic pregnancy, and a second opinion was sought before starting methotrexate treatment.

She had her first dose of methotrexate at 50 mg/m^2^. On day four post methotrexate administration, β-hCG level was 13,933 IU/L, and on day seven, it was 13,307 IU/L. As the drop was less than 15%, she was offered a second dose of methotrexate; however, she declined.

A plan was made to continue to monitor the β-hCG level. On day 10, the β-hCG level dropped to 10,194 IU/L and continued to drop markedly over the following days.

**Figure 2:**
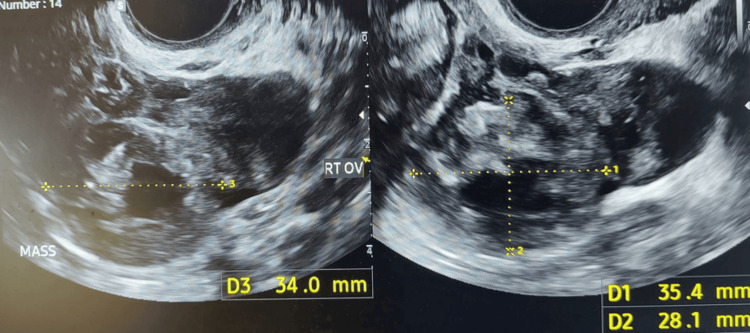
Ectopic pregnancy mass measuring 34 x 35.4 x 28.1 mm.

On day 25 post treatment, when the β-hCG level reached 941 IU/L and the patient was haemodynamically stable, discharge from the hospital with planned outpatient follow-up was discussed. She started to feel a sharp intermittent right iliac fossa pain with no change in her blood test results, and a transvaginal scan (Figure 2) showed a slight increase in mass size, with no evidence of rupture. Simultaneously, a urinary tract infection was diagnosed, and a course of antibiotics was given.

The β-hCG level continued to drop, reaching 76 IU/L on day 40 post treatment, with gradually reducing pain. The patient was discharged home with an appointment for repeat β-hCG and transvaginal scan a week later.

**Figure 3:**
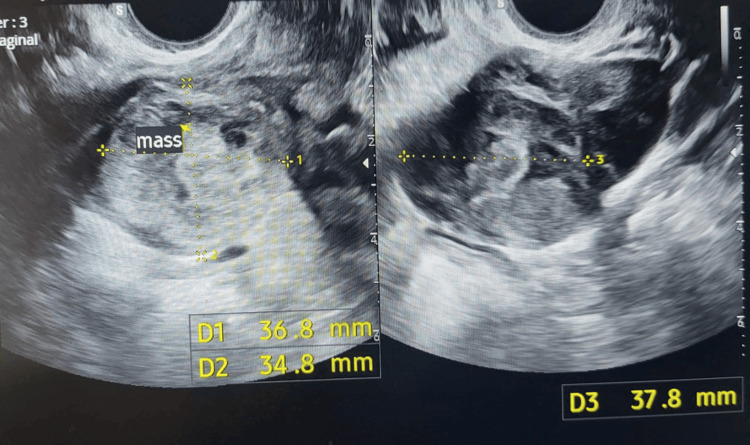
Ectopic pregnancy mass measuring 36.8 x 34.8 x 37.8 mm.

On her first outpatient follow-up appointment, the patient reported that the pain had markedly improved. The β-hCG level was 26 IU/L, and the adnexal mass was reported (Figure 3).

**Figure 4:**
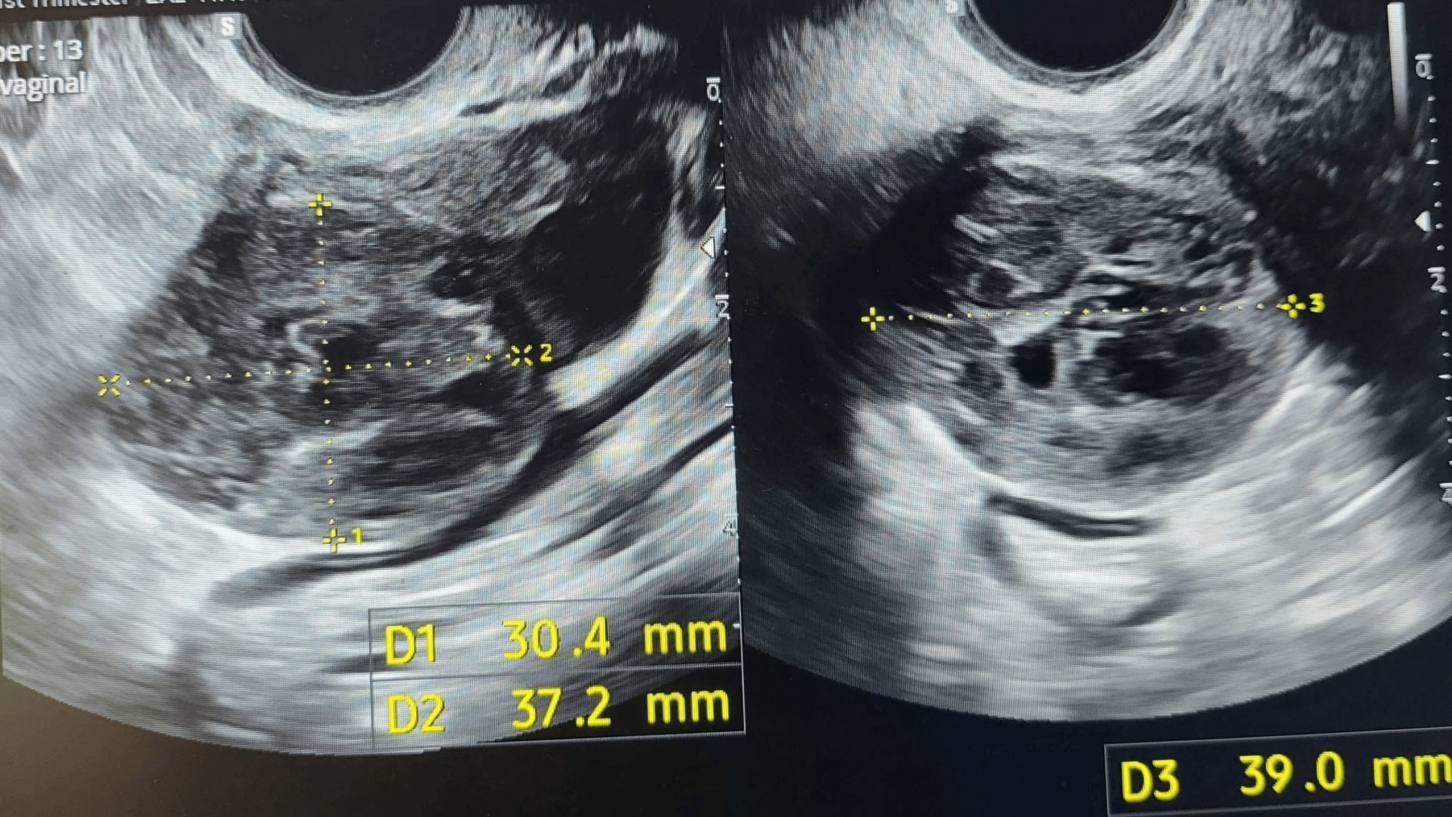
Ectopic pregnancy measuring 30 x 37 x 39 mm.

Four days later, the patient attended the early pregnancy unit complaining of mild vaginal bleeding and crampy lower abdominal pain. She was stable with a β-hCG level of 4 IU/L. Three days later (day 54 post treatment), she reported having her normal period. A follow-up scan was done one week later (day 61 post treatment), which reported no change in the adnexal mass size (Figure 4).

## Discussion

Sowter et al. (2001) concluded from their prospective randomised trial, comparing a single dose of methotrexate vs. laparoscopic surgery for the management of unruptured tubal ectopic, that at serum β-hCG concentrations over 1500 IU/L, women are likely to need more than one dose of methotrexate and a significant proportion will require surgical intervention [9]. The inclusion criteria for their study were a serum β-hCG concentration below 5000 IU/L, an adnexal mass less than 3.5 cm in diameter with absent fetal heart activity, and minimal haemoperitoneum on transvaginal ultrasound (estimated to be less than 300 mL).

The National Institute for Health and Care Excellence (NICE) clinical guidance on the management of ectopic pregnancy and miscarriage, published in 2012, after investigating the evidence from 10 randomised trials, recommended that “methotrexate only be offered as a first line treatment for women with an adnexal mass smaller than 35 mm and β-hCG level of less than 1500 IU/l” [10].

The NICE Guideline Development Group (GDG), considering the evidence from the above study, suggested that with β-hCG levels between 1500 IU/l and 5000 IU/l, it would still be appropriate to offer medical management, acknowledging the fact that further intervention could be required [10].

The NICE guideline updates in 2019 and 2023 continued to adopt the same recommendations after reviewing the evidence from four randomised controlled trials with varied ultrasound findings and pre-treatment β-hCG. All the included studies had pre-treatment β-hCG levels of less than 5000 IU/l, varying from 1000 to 5000 IU/l [11].

The Practice Committee of the American Society for Reproductive Medicine, regarding medical treatment of ectopic pregnancy, demonstrated a positive correlation between successful medical treatment and lower initial β-hCG level, acknowledging that there was no consensus on the best predictive threshold value for success or failure [8]. The committee reviewed the reported failure rates of different studies that used methotrexate for the treatment of ectopic pregnancies with high initial β-hCG levels. Lipscomb et al. (1999) reported a failure rate with single-dose treatment of 13% with pre-treatment β-hCG level between 5000 IU/L and 9999 IU/L. The failure rate increased to 18% when the β-hCG level was between 10,000 IU/L and 14,999 IU/L [12]. Elito et al. (1999) reported a higher failure rate of 57.2% when the initial β-hCG level was above 5000 IU/L [13]. Potter et al. (2003) found comparable results with failure rates of 62% with β-hCG levels above 5000 IU/L. Of note, the first study stratified the failure rates by the initial β-hCG levels, which could be the reason for the lower reported failure rate, while the latter two studies reported the joint failure rate for all patients with initial β-hCG levels of more than 5000 IU/L, without stratification, with single-dose treatment [14]. The committee concluded an odds ratio (OR) for failure of 5.45, after analysis of combined published data, when initial β-hCG values were above 5000 IU/L compared to concentrations that were below that threshold [8].

The American College of Obstetricians and Gynecologists (ACOG) considered the initial β-hCG level higher than 5000 IU/L as a relative contraindication, not an absolute cut-off, for methotrexate treatment. The guideline considered the evidence from a systematic review showing a failure rate of 14.3% when initial β-hCG was more than 5000 IU/L compared to 3.7% when β-hCG was less than 5000 IU/L [3].

A retrospective cohort study of 184 patients with diagnosed unruptured tubal ectopic pregnancy who received methotrexate medical treatment (single-dose treatment for those with an initial β-HCG < 4800 IU/L and the double-dose regimen for those with an initial β-HCG > 4800 IU/L) showed a 92.6% success rate in the single dose group, and 81.3% in the double dose group, without serious adverse effects. The study concluded that methotrexate as a first-line treatment for tubal ectopic pregnancy with β-HCG > 2000 IU/L is promising [15].

Medical management could be a cost-effective option that avoids the risk of morbidity associated with surgery and anaesthesia [16]. However, the expected time for resolution, with an initial high HCG level, makes surgical management in these cases a more cost-effective option [3].

Additionally, a higher failure rate means not only increased follow-up cost, but more importantly, higher chances for patients to experience tubal rupture, a leading cause of maternal deaths from ectopic pregnancy [2]. The guidelines aim to ensure safe and evidence-based practices. On the other hand, respecting patient autonomy, when choices and risks are explained, is a mandatory ethical practice. Consequently, could methotrexate be an option for women with an initial high HCG level, who find surgical management unacceptable, provided that they have accurate information and appropriate counselling on increased failure rate and risk of rupture with careful case selection and intensive monitoring?

In maternity services, the NHS Long Term Plan made commitments to deliver choice and personalised care. Informed decision-making is a central part of personalised care and support planning [17], where patient-centred care and autonomy are key. For example, birth choices clinics are offered by some NHS organisations for pregnant women who decline recommended treatment or ask for care that goes outside of local/national maternity guidelines. Birth choices clinics provide more senior support, discussion, informed decision, and personalised care [18].

We are uncertain about the significance of a persistent ectopic mass after methotrexate treatment. The literature review yielded a paucity of data regarding the persistent tubal ectopic pregnancy mass after successful methotrexate treatment. One case reported chronic pelvic pain for nearly seven months after successful medical management of ectopic pregnancy until definitive surgical management for removal of the persistent ectopic mass was performed [19].

## Conclusions

Surgical management of ectopic pregnancy is cost-effective when the time to resolution is expected to be long, as in the case of an initial high HCG level.

Respecting patient autonomy, when choices and risks are informed, is a mandatory ethical practice. Challenges for clinicians to provide treatment outside recommended standard practice, especially when physicians perceive this as an unsafe practice, need to be addressed in national and legal guidance. Evidence on the long-term complications, for example, long-term fertility impact and tubal patency and function, of persistent ectopic mass after medical treatment is essential for robust counselling on risks.
